# Gut-muscle axis crosstalk in age-related sarcopenia: mechanisms and therapeutic targets

**DOI:** 10.3389/fmicb.2025.1638880

**Published:** 2025-12-17

**Authors:** Ling-Li Gao, Yan Chen, Ting Dai, Jie Zheng, Shuo-Shuo Su, Yi-Xun Chen, Li-Dian Chen, Jing Gao, Xiao-Dong Feng

**Affiliations:** 1 Department of Rehabilitation, The First Affiliated Hospital of Henan University of Chinese Medicine, Zhengzhou, China; 2 School of Rehabilitation Medicine, Henan University of Chinese Medicine, Zhengzhou, China

**Keywords:** gut-muscle axis, crosstalk, age-related sarcopenia, inflammation, microbial metabolites, neuroendocrine system, myokines, therapeutic targets

## Abstract

The interplay between gut microbiota and sarcopenia has emerged as a cutting-edge research topic in the medical field, garnering significant attention. Sarcopenia is an age-related syndrome characterized by a progressive decline in skeletal muscle mass, strength, and function, which profoundly impacts the quality of life in older adults and imposes substantial socioeconomic burdens on many counties. Accumulating evidence indicates that alterations in the gut microbiota are not only linked to various intestinal disorders but also to aging-associated conditions, such as sarcopenia. The gut microbiota plays a pivotal role in regulating skeletal muscle homeostasis via its metabolic products and is increasingly recognized as a potential pathophysiological factor contributing to sarcopenia development. Skeletal muscle, functioning as both a motor and endocrine organ, secretes myokines that exert critical regulatory effects on the gut microbiota. In sarcopenic individuals, reduced secretion of myokines correlates with decreased microbial diversity and compositional shifts, marked by diminished beneficial microbes and increased potentially harmful species. This establishes a vicious cycle of gut dysbiosis-sarcopenia-gut dysbiosis. Modulation of the gut microbiota has been demonstrated to enhance muscle mass and function in elderly patients with sarcopenia. Metabolites derived from the gut microbiota, such as amino acids, lipopolysaccharides, and short-chain fatty acids, are known to modulate skeletal muscle protein metabolism by influencing anabolic and catabolic pathways. Nevertheless, the bidirectional mechanisms underlying the relationship between gut microbiota and age-related sarcopenia remain incompletely understood. In this review, we aim to: (1) integrate current knowledge regarding the bidirectional interaction between sarcopenia and gut microbiota; (2) summarize existing management strategies for age-related sarcopenia based on this interaction.

## 1 Introduction

Sarcopenia is a systemic and progressive disorder of skeletal muscle characterized by an age-related decline in muscle mass, strength, and/or function (Sayer et al., 2024). It is estimated that approximately 10%–16% of older adults worldwide are affected by sarcopenia, with a prevalence ranging from 5.5% to 25.7% in Asian countries (Chen et al., 2020; Yuan and Larsson, 2023). Sarcopenia increases vulnerability to adverse outcomes such as heightened risk of falls and mortality, decline in physical function, and frailty, which significantly impacts the quality of life for the elderly population. Reports indicate that patients with sarcopenia face a higher risk of hospitalization and incur greater hospital costs compared to their non-sarcopenic counterparts(Kelley and Kelley, 2017). Despite its clinical significance, the mechanisms underlying sarcopenia remain incompletely understood. Emerging evidence highlights the gut microbiota as a critical extrinsic regulator of skeletal muscle homeostasis, unveiling a novel gut-muscle axis that may drive sarcopenia pathogenesis.

The gut microbiota constitutes a complex and dynamic ecosystem, comprising trillions of microorganisms spanning bacteria, archaea, fungi, and viruses (Barry et al., 2024). This microbial consortium exerts profound physiological impacts, modulating host nutrient metabolism, immune homeostasis, and intestinal barrier integrity through intricate host-microbe crosstalk (Ma and Lee, 2025). Notably, interindividual variability in microbial composition arises from heterogeneous factors, including dietary patterns, host age and lifestyle determinants (Olvera-Rosales et al., 2021). The gut microbiota is dominated by Bacteroidetes and Firmicutes (collectively representing 90% of the healthy adult microbiota), with subordinate contributions from Actinobacteria, Proteobacteria, and Verrucomicrobia (Almheiri et al., 2025; Gomaa, 2020; Human Microbiome and Project Consortium, 2012). Key health-promoting genera such as Bifidobacterium, Akkermansia, and Lactobacillus demonstrate pleiotropic benefits, including vitamin synthesis such as vitamin K and vitamin B12, dietary fiber fermentation into short chain fatty acids (SCFAs), and reinforcement of mucosal barrier function via tight junction regulation(Elahi et al., 2023; Si et al., 2022). Clinically, microbiota dysbiosis, characterized by reduced microbial diversity, depletion of commensals, and expansion of pathobionts, has been mechanistically linked to multiple pathologies. Emerging evidence implicates dysbiosis in neurological disorders, cardiovascular disease, inflammatory bowel diseases, and sarcopenia (Chen et al., 2021).

Accumulating evidence indicates a distinct dysbiosis in the gut microbiome of individuals with sarcopenia. One of the most prominent alterations is a decreased Firmicutes/Bacteroidetes (F/B) ratio, characterized by a 10%–20% reduction in Firmicutes abundance and an 8%–15% increase in Bacteroidetes. Further analysis demonstrates a marked depletion of beneficial short-chain fatty acid (SCFA)-producing genera, including Faecalibacterium, prausnitzii and Roseburia, with average reductions ranging from 25% to 40%. In contrast, multiple potential pathobionts, particularly members of the Enterobacteriaceae family, exhibit several-fold increases in abundance (Lee et al., 2022). This microbial ecological imbalance, defined by the loss of symbiotic taxa and expansion of pro-inflammatory species, represents a hallmark feature of the gut microbiome in sarcopenia. The depletion of these beneficial microbial communities’ results in a significant reduction in the intestinal production of SCFAs, particularly butyrate. Butyrate serves not only as a primary energy substrate for colonic epithelial cells but also enters systemic circulation, where it promotes muscle protein synthesis and tissue repair through the inhibition of histone deacetylases (HDACs) in skeletal muscle. Consequently, the diminished abundance of SCFA-producing bacteria disrupts this critical signaling axis essential for maintaining muscle homeostasis. In contrast, the abundance of pro-inflammatory bacteria Escherichia-Shigella is increased(Kang et al., 2021). In an experimental animal model of sarcopenia, rats exhibited increased Escherichia-Shigella abundance, elevated plasma LPS concentrations, activation of the TLR4/MyD88/NF-κB signaling pathway, up-regulation of MuRF-1 and Atrogin-1 expression, and a reduction in muscle fiber cross-sectional area (CSA) (Lahiri et al., 2019). A bidirectional two-sample Mendelian randomization analysis has substantiated causal relationships between specific gut microbial features and musculoskeletal disorders. The study revealed that gut microbiota dysbiosis likely contributes to sarcopenia pathogenesis through both inflammatory cascades and metabolic dysfunction pathways (Chen et al., 2023). Notably, preclinical studies utilizing fecal microbiota transplantation (FMT) from young to aged murine models have reported significant improvements in muscle mass (*p* < 0.01) and physical performance metrics, with grip strength increasing by approximately 30%–50% (Kim et al., 2022). In preliminary clinical investigations, a single FMT via colonoscopy from healthy young donors in elderly patients with sarcopenia was associated with a reduction in IL-6 and TNF-α levels, along with a significant increase in the abundances of Faecalibacterium, Roseburia, and Prevotella. These changes coincided with a 60% decrease in Enterobacteriaceae, a 25% decrease in serum zonulin, and a 20% decrease in LPS-BP. Additionally, improvements in muscle mass, strength, and physical function were observed, and the therapeutic effects appeared to be sustained for up to 24 weeks. No colonization by multidrug-resistant bacteria was observed (Yang et al., 2025). On the other hand, signals originating from skeletal muscle can also modulate the intestinal microenvironment. Skeletal muscle atrophy or functional impairment leads to reduced secretion of beneficial myokines, such as irisin, thereby potentially exacerbating intestinal barrier dysfunction and microbial dysbiosis (Luo et al., 2023). Thus, the relationship between gut microbiota imbalance and muscle degeneration is not unidirectional, but rather constitutes a bidirectional, self-reinforcing vicious cycle that collectively drives the pathological progression of sarcopenia.

This review synthesizes current evidence on the bidirectional mechanisms linking gut microbiota dysbiosis to sarcopenia pathogenesis, evaluates evidence-based interventions targeting the gut-muscle axis in aging populations. Elucidating the crosstalk between microbial metabolites and myocellular signaling pathways is pivotal for developing microbiota-centric therapeutics against age-related muscle deterioration.

## 2 Sarcopenia: key mechanisms and contributing factors

### 2.1 Inflammaging

During the aging process, chronic sterile inflammation is characterized by a mild yet persistent increase in pro-inflammatory factor levels, a phenomenon referred to as inflammaging (Antuña et al., 2022). Inflammaging influences the progression of sarcopenia through multiple mechanisms, such as cellular senescence, mitochondrial DNA damage and changes in adipose tissue and (Liang et al., 2022).

Cellular senescence leads to the secretion of a senescence-associated secretory phenotype (SASP), a complex of pro-inflammatory cytokines, chemokines, proteases, and growth factors (Hernandez-Segura et al., 2018). Pro-inflammatory factors, such as tumor necrosis factor-alpha (TNF-α), activate the ubiquitin-proteasome system (UPS) and autophagy signaling pathways, thereby promoting catabolic processes, while simultaneously inhibiting the PI3K/Akt/mTORC signaling pathway, which reduces protein synthesis (Webster et al., 2020). Upon binding to tumor necrosis factor receptor 1 (TNFR1), TNF-α recruits tumor necrosis factor receptor type 1-associated death domain protein andTNF receptor-associated factor 2/5, leading to the activation of the IκB Kinase (IKK) complex. IKK phosphorylates IκBα, resulting in its ubiquitination and degradation, and subsequently releasing nuclear factor kappa light chain enhancer of activated B cells (NF-κB) (Li et al., 2016). NF-κB binds to the promoter regions of muscle ring-finger protein 1 (MuRF1) and muscle atrophy f-box protein(Atrogin-1), upregulates their transcription (Schiaffino et al., 2013), accelerates protein degradation. Additionally, TNF-α activates jun n-terminal kinase, inhibits insulin receptor substrate 1, and blocks the PI3K/Akt signaling pathway, leading to the dephosphorylation of fork head box O3 (FoxO3) (Ko et al., 2023). Dephosphorylated FoxO3 translocates to the nucleus and directly binds to the promoters of MuRF1 and Atrogin-1, promoting their transcription. Furthermore, dephosphorylated FoxO3 upregulates the expression of Microtubule-associated protein light chain 3(LC3)and Beclin-1, stimulating the formation of autophagosomes (Zhang et al., 2023). In aged mice, geriatric satellite cells switch from reversible quiescence to p16^∧^INK4a-driven senescence, secreting IL-6 and TNF-α (SASP) and leading to a 25 % reduction in myofiber cross-sectional area; genetic or pharmacological suppression of p16^∧^INK4a or JAK/STAT signaling restores muscle regeneration (Sousa-Victor et al., 2014).

Transforming growth factor-β (TGF-β), as a critical component of the SASP, exerts significant influence on tissue repair, fibrotic processes, and aging-related mechanisms (Wang et al., 2024). TGF-β inhibits the proliferation and differentiation of muscle stem cells via the p38 Mitogen-Activated Protein Kinase signaling pathway, hindering muscle regeneration (Kim and Lee, 2017). TGF-β also activates autophagy-related genes such as LC3 and BCL2/adenovirus E1B 19kDa interacting protein 3 (BNIP3) through the smad3 signaling pathway, leading to excessive autophagy and myofiber damage (Kim and Lee, 2017). With advancing age, adipose tissue accumulates ectopically within skeletal muscle, leading to intramuscular fat infiltration (Liang et al., 2022), which is a key characteristic of sarcopenia (Al Saedi et al., 2022). Intermuscular adipose tissue (IMAT) contributes to insulin resistance and inflammation (Beasley et al., 2009). Studies have demonstrated that increased thigh IMAT is associated with an elevated risk of type 2 diabetes and impaired glucose tolerance (Al Saedi et al., 2022). Additionally, higher levels of thigh IMAT are significantly correlated with increased Interleukin-6 (IL-6) and C-reactive Protein (CRP) levels in men (Beasley et al., 2009).

### 2.2 Mitochondrial dysfunction

Mitochondrial dysfunction is a central pathological mechanism in age-related sarcopenia, characterized by progressive age-related declines in bioenergetic efficiency, leading to impaired ATP synthesis and compromised muscle protein anabolism (Kamarulzaman and Makpol, 2025). A decrease in the expression of the master regulator of mitochondrial biogenesis, peroxisome proliferator-activated receptor-gamma coactivator 1 alpha (PGC-1α), has been reported in age-related sarcopenia. Clinical studies demonstrate a significant association between circulating PGC-1α concentrations and gait speed in older adults (Joseph et al., 2012). Experimental models further reveal that genetic upregulation of PGC-1α in injury-induced muscle atrophy models sustains mitochondrial density and contractile force (Southern et al., 2019). Furthermore, endurance training-induced mitigation of age-related mitochondrial dysfunctionis mediated through PGC-1α-dependent mechanisms, as evidenced by the complete abolition of these protective effects in PGC-1α−/− murine models (Leick et al., 2010). Furthermore, the findings reveal that in aged wild-type (WT) mice, PGC-1α expression was upregulated two-fold following training, accompanied by a 42% improvement in treadmill endurance. In contrast, knockout (KO) mice (mPGC-1α−/−) exhibited neither an increase in PGC-1α expression nor any functional improvement. Specifically, the time to exhaustion during treadmill testing in the WT training group increased by 42% (*P* < 0.01), whereas the KO training group showed only an 8% increase, which was not statistically significant. These results provide strong evidence that the enhancement of endurance resulting from exercise training is causally linked to PGC-1α upregulation and mitochondrial remodeling (Leick et al., 2010).

Mitophagy is essential for maintaining mitochondrial integrity, and its dysregulation exacerbates sarcopenia. Previous studies have highlighted the critical role of mitophagy in preventing disuse muscle atrophy. Examination of immobilized mt-Keima mice revealed significant decreases in muscle strength and atrophy in type IIA, IIX, and IIB muscle fibers, accompanied by a notable increase in mitophagy flux. Inhibition of mitophagy flux with colchicine exacerbated atrophy in type IIX and IIB fibers, along with a marked reduction in mitochondrial function and activation of apoptosis-related proteins CASP9 and CASP3 (Rahman et al., 2025). Mitophagy in mammals is regulated by PTEN-induced kinase 1 (PINK1) and parkin (Liu et al., 2021). Deletion of parkin reduces muscle mass and leads to poor physical function in older mice, while overexpression of parkin improves skeletal muscle function in older mice (Liu et al., 2021). Genetic deletion of PINK1 and parkin leads to dysfunction of mitophagy and mitochondria, muscle destruction, while increased mitophagy with overexpression of PINK1, parkin, and dynamin-related protein 1 in drosophila reduces age-related muscle dysfunction (Romanello, 2020). Since parkin and PINK1 regulate mitophagy and mediate sarcopenia-related muscle weakness, increases in the expression of these proteins may likely be a useful strategy to form a healthy mitochondrial network in skeletal muscles.

### 2.3 Degeneration of the neuromuscular junction

The etiology of sarcopenia is multi-factorial, with deterioration of neuromuscular junction (NMJ) as one of the major causes (Bao et al., 2020). The integrity and function of the NMJ, bridging the gap between the nervous and muscular systems, are pivotal for muscle strength input and reliable neural control. Consequently, NMJ has long been a focal point in researching skeletal muscle dysfunction and sarcopenia during the aging process (Arnold and Clark, 2023). A study examining the fidelity of NMJ transmission in hind limb muscles of male and female C57BL/6J mice across various ages revealed that NMJ transmission failure primarily manifests during the aging phase of mice, leading to reduced excitability of muscle fibers in these mice (Chugh et al., 2020). Furthermore, NMJ defects are closely correlated with hind limb grip strength, gastrocnemius muscle weight, loss of peak contractile torque, and loss of motor units (Padilla et al., 2021). Evidence from electrophysiological studies indicates age-related impairments in neuromuscular junction (NMJ) stability. Sarto et al. (2024) reported that, compared with young individuals, older adults exhibited a significant increase in near-fiber jitter on electromyography, suggesting a decline in conduction stability at the NMJ. These electrophysiological changes were accompanied by elevated levels of the C-terminal agrin fragment (CAF), alterations in caveolin-3 expression, and increased serum neurofilament light chain (NfL). Collectively, these neuromuscular alterations were associated with concomitant muscle atrophy and weakness (Sarto et al., 2024). Up porting the functional relevance of these observations, a longitudinal cohort study by Gilmore et al. (2017) further observed that near-fiber jitter increased by approximately 31%–43% during the progression from pre-sarcopenia to severe sarcopenia. Together, these findings suggest that NMJ conduction instability may serve as a sensitive biomarker for assessing sarcopenia severity.

The NMJ, serving as a critical synapse linking motor neurons and muscle fibers, undergoes progressive degeneration during aging. This degeneration is primarily characterized by structural deterioration, alterations in synaptic transmission function, and diminished plasticity, ultimately contributing to the decline in muscle mass and functional capacity. With advancing age, the presynaptic structure of the NMJ exhibits axonal denervation, reinnervation, and remodeling, along with changes in intramuscular nerve branching patterns (Iyer et al., 2021). In aged rodents, the NMJ becomes increasingly fragmented, marked by reduced synaptic folds, decreased numbers of presynaptic vesicles and nerve terminals, dispersed motor endplate regions, reduced density of acetylcholine receptors (AChRs), and diminished binding affinity of AChRs. These structural and functional changes may impair synaptic transmission efficiency, leading to denervation-induced muscle dysfunction and morphological alterations at the NMJ, such as increased endplate area, reduced synaptic folds, and decreased nerve terminal branches. Such modifications are likely to compromise the normal function of the NMJ. Factors such as reduced release of acetylcholine from presynaptic terminals and decreased sensitivity of postsynaptic membranes to AChRs further contribute to the decline in synaptic transmission efficiency, weakening the signaling for muscle contraction and resulting in reduced muscle strength (Taetzsch and Valdez, 2018). Although the NMJ possesses a degree of intrinsic plasticity that allows adaptive remodeling in response to neuromuscular diseases or injuries, this capacity diminishes with age, rendering the NMJ more susceptible to damage.

In recent years, the agrin/MuSK/LRP4 signaling pathway has been identified as one of the key regulatory mechanisms governing NMJ development and stability (Zong et al., 2012). Agrin, a glycoprotein secreted by motor neurons and a pivotal factor in NMJ formation, binds to low-density lipoprotein receptor-related protein 4 (LRP4) on the muscle membrane. This interaction activates muscle-specific kinase (MuSK), initiating a cascade of downstream signaling events that promote the clustering of AChRs on the postsynaptic membrane, thereby completing NMJ formation (Zong and Jin, 2012). Dysregulation or abnormalities in this signaling pathway may result in structural and functional impairments of the NMJ, potentially leading to neuromuscular disorders such as myasthenia gravis (Walker et al., 2021). Lahiri et al. (2019) observed that the gene expressions of acetylcholine receptor (AChR) subunits (Chrna1, Chrnb1, Chrne, Chrnd) and essential assembly factors, including Rapsyn and Lrp4, were significantly downregulated in the tibialis anterior muscle of germ-free (GF) mice, suggesting a potential impairment in AChR assembly. These alterations were reversed following colonization with conventional microbiota (C-GF). Furthermore, GF mice exhibited reduced gene expression of troponin, decreased grip strength, as well as diminished locomotor and upright activities (*P* < 0.05 to *P* < 0.001) (Lahiri et al., 2019). Collectively, these findings indicate that the absence of gut microbiota compromises neuromuscular junction (NMJ) function and muscle strength through disruption of AChR assembly and suppression of downstream contractile protein expression. Shi et al. (2024) conducted bidirectional two-sample Mendelian randomization (MR) and mediation analysis to suggest that elevated levels of Enterococcus faecium and reduced levels of *Faecalibacterium prausnitzii* are associated with a significantly increased risk of myasthenia gravis, mediated through decreased serum agrin concentrations and disruption of the agrin-MuSK-LRP4 signaling pathway. This pioneering study establishes, for the first time, the “gut microbiota–neuromuscular junction” causal axis at the human genome-metagenome interface, offering a mechanistic foundation for microbiota-targeted interventions in neuromuscular junction (NMJ)-related disorders.

### 2.4 Neuroendocrine dysregulation

The neuroendocrine system plays a core role in the occurrence and development of sarcopenia, influencing muscle mass and function through regulating muscle protein synthesis and degradation, energy metabolism, and inflammatory responses.

Growth hormone (GH), secreted by the anterior pituitary gland, stimulates the liver to synthesize growth factor-1(IGF-1) (Gharahdaghi et al., 2020), which promotes muscle protein synthesis by activating the PI3K/Akt/mTOR pathway. With aging, the secretion levels of GH and IGF-1significantly decline, leading to reduced muscle synthesis and atrophy (Hage and Salvatori, 2023). Research findings suggest that serum IGF-I levels in growth hormone (GH)-deficient (lit/lit) mice decline progressively with age and are more than fourfold lower than those in normal (lit/+) littermates, which maintain stable IGF-I levels up to 52 weeks. This is accompanied by a reduction in total IGF binding capacity in lit/lit mice, primarily due to decreased levels of IGF binding proteins, particularly IGFBP-3. Notably, a significant age-related reduction in protein percentage was observed exclusively in GH-deficient mice (Donahue and Beamer, 1993). The Snell dwarf mouse demonstrates a significantly higher myonuclear density in the soleus muscle compared to normal mice; however, the total number of nuclei is reduced relative to normal mice(Stickland et al., 1994). This phenomenon can be ascribed to the diminished secretion of growth hormone resulting from pituitary dysfunction, which consequently impairs muscle synthesis and development.

Sex hormones, such as testosterone and estrogen, are also crucial for maintaining muscle mass and strength. With aging, the levels of sex hormones gradually decline, leading to a reduction in muscle mass and strength. Testosterone promotes muscle protein synthesis and inhibits proteolysis primarily by activating androgen receptors in myonuclei and satellite cells. It also modulates key metabolic factors such as GH and IGF-1, which are essential for muscle repair and regeneration (Shigehara et al., 2022). Conversely, low testosterone levels exacerbate chronic inflammation and oxidative stress, leading to activation of the ubiquitin-proteasome system (UPS) and accelerated muscle protein degradation (Parahiba et al., 2020). Clinically, the reduction in testosterone levels in elderly men is closely associated with decreased muscle mass and strength, and testosterone supplementation has been shown to improve grip strength, leg strength, and overall physical function in sarcopenic individuals (Parahiba et al., 2020; Shigehara et al., 2022). Similarly, estrogen also plays an important role in maintaining muscle mass and function in women, and its decline can lead to muscle deterioration. Estradiol promotes beneficial effects on skeletal muscle by stimulating satellite cell proliferation and limits inflammatory stress-induced damage to skeletal muscle (Geraci et al., 2021). Studies have demonstrated that postmenopausal women experience a decline in estrogen levels, which contributes to reduced skeletal muscle function and increases the risk of sarcopenia (Lu and Tian, 2023).

**FIGURE 1:**
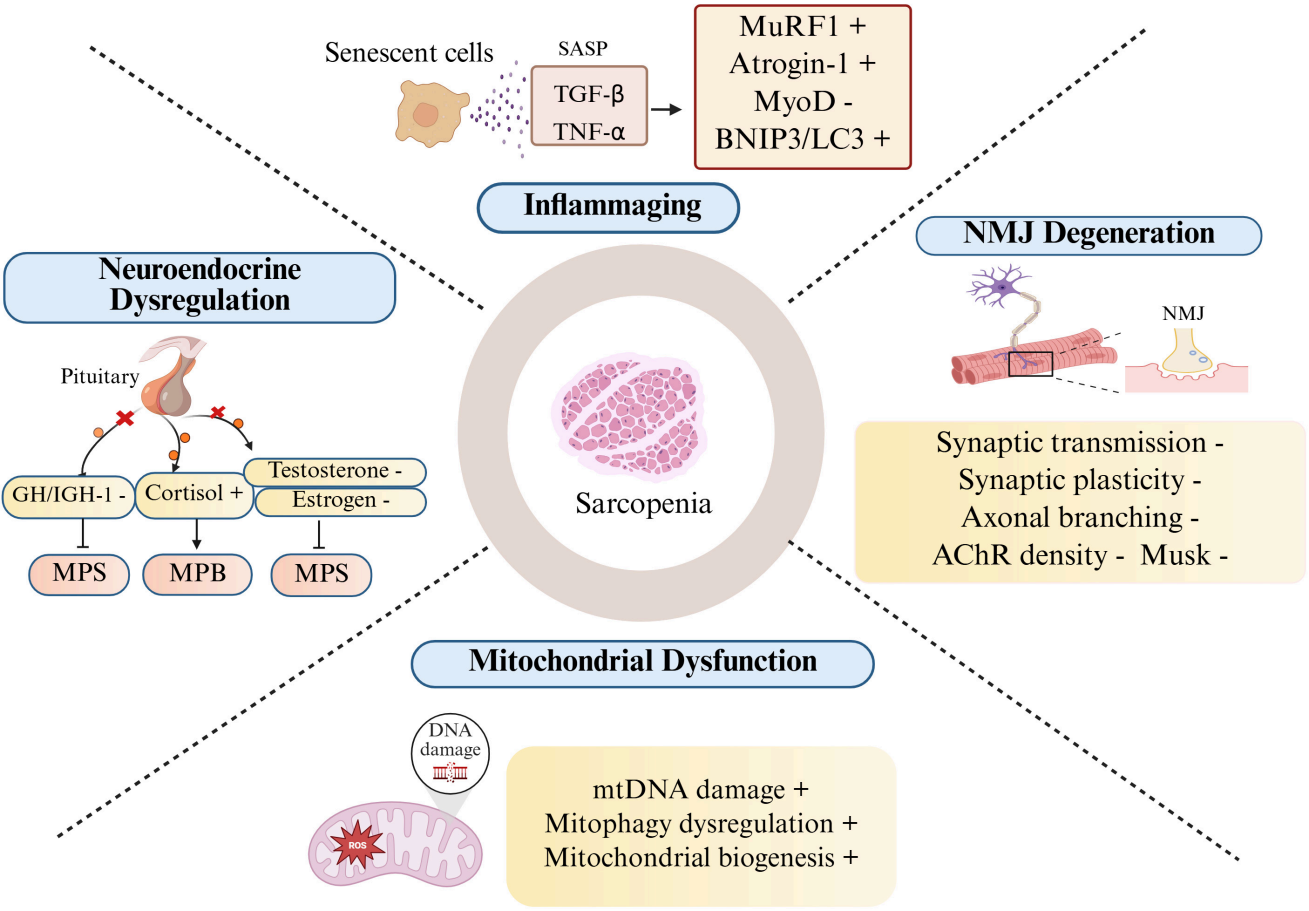
Sarcopenia: key mechanisms and contributing factors. (Created in BioRender. Lingli, G. (2025) https://BioRender.com/undefined). Aging drives the progression and development of sarcopenia via chronic inflammation, alterations in NMJ integrity, mitochondrial dysfunction, and neuroendocrine dysregulation. (1) Inflammaging: The accumulation of senescent cells and their secretion of SASP factors, such as TGF-β and TNF-α, upregulates key mediators of muscle atrophy. This includes the E3 ubiquitin ligases MuRF1 and Atrogin-1 (promoting proteolysis via the UPS) and proteins such as BNIP3 and LC3 (activating autophagy). Concurrently, the expression of the myogenic transcription factor MyoD is suppressed, impairing muscle regeneration (R) (Webster et al., 2020). (2) NMJ degeneration: Aging is associated with progressive structural and functional decline at the NMJ. Key features include presynaptic axonal branching loss, deficits in synaptic transmission (e.g., reduced AChR, density and MuSK, signaling), and impaired synaptic plasticity. These alterations disrupt neuromuscular signaling, leading to muscle denervation and atrophy (P/C) (Iyer et al., 2021; Lahiri et al., 2019; Sarto et al., 2024). (3) Mitochondrial dysfunction: mtDNA damage, mitophagy dysregulation, and diminished biogenesis (PGC-1α-) compromise energy metabolism, further contributing to muscle weakness (P) (Rahman et al., 2025; Southern et al., 2019). 4) Neuroendocrine dysregulation: Aging alters the hormonal milieu in a manner that favors catabolism. This is characterized by a decline in anabolic hormones (GH; IGF-1; testosterone; estrogen) coupled with hyperactivity of the hypothalamic-pituitary-adrenal (HPA) axis and elevated cortisol. This collective shift suppresses MPS and promotes MPB, leading to net muscle loss (P/C) (Donahue and Beamer, 1993; Hage and Salvatori, 2023; Schakman et al., 2008; Shigehara et al., 2022). +, increase; –, decrease; ⊥, inhibition. (C), Clinical; (P), Preclinical; (R): Review. SASP, senescence-associated secretory phenotype; UPS, ubiquitin-proteasome system; NMJ, neuromuscular junction; AChR, acetylcholine receptor; MPS, muscle protein synthesis; MPB, muscle protein breakdown; HPA, hypothalamic-pituitary-adrenal.

Aging-related excessive activation of the hypothalamic-pituitary-adrenal (HPA) axis results in elevated cortisol levels. Glucocorticoids bind to glucocorticoid receptors in skeletal muscle, thereby activating the UPS and autophagy-lysosomal pathway, which promotes muscle protein degradation (Schakman et al., 2008). Furthermore, chronic low-grade inflammation associated with HPA axis activation, characterized by increased levels of IL-6 and TNF-α as well as oxidative stress, exacerbates muscle damage (Manoli et al., 2005). Pro-inflammatory cytokines inhibit the IGF-1/PI3K/Akt signaling pathway, leading to reduced muscle protein synthesis. Excessive HPA axis activity suppresses the GH-IGF-1 axis, a critical pathway for maintaining muscle mass and satellite cell activity (Schakman et al., 2008). The age-related decline in GH and IGF-1 levels, coupled with HPA axis-driven cortisol elevation, creates a “catabolic-anabolic imbalance,” resulting in net muscle loss. Studies have demonstrated that physical performance in older adults is negatively correlated with elevated cortisol levels (Peeters et al., 2007; Figure 1).

## 3 Gut microbiota dysbiosis in sarcopenia

Accumulating evidence indicates that patients with sarcopenia exhibit a distinct gut microbiota dysbiosis profile, which may serve as a microbiological foundation underlying the onset and progression of this condition. At the phylum level, a hallmark alteration is the reduced Firmicutes/Bacteroidetes (F/B) ratio, typically indicative of diminished relative abundance of metabolically active microbial communities (Mayer et al., 2024).

At the genus level, cross-sectional comparisons (sarcopenia patients vs. healthy controls) and longitudinal association analyses (linking microbial abundance to muscle function parameters) have identified several bacterial taxa with potential diagnostic or mechanistic significance (Mayer et al., 2024; Zhu et al., 2024). Notably, there is marked depletion of beneficial bacteria: core short-chain fatty acid (SCFA)-producing genera, such as *Faecalibacterium*, *Prevotella*, and *Lachnoclostridium*, are consistently observed at lower abundances in individuals with sarcopenia. Mendelian randomization studies further support a causal relationship, suggesting that higher abundance of *Faecalibacterium* is positively associated with appendicular skeletal muscle mass (ALM) and walking speed (WS) (Zhang et al., 2024). Concurrently, an expansion of potential opportunistic pathogens is frequently observed in the gut microbiota of sarcopenia patients, including elevated levels of *Bacteroides*, *Parabacteroides*, and *Shigella* (Han et al., 2022). Moreover, additional genera negatively correlated with muscle performance, such as Megamonas, as well as those linked to grip strength (GS), including Porphyromonadaceae and Terrisporobacter, collectively contribute to a complex and disease-specific microbial network.

In summary, sarcopenia-associated gut dysbiosis manifests as a dual-track pattern, characterized by: (i) a reduction in symbiotic bacteria that possess anti-inflammatory and SCFA-producing capabilities; and (ii) an increase in pro-inflammatory or functionally uncharacterized taxa. This unique microecological configuration not only clarifies the potential pathways through which gut microbes influence muscle physiology, via metabolic signaling and immune modulation, but also underscores the reciprocal regulation of the intestinal ecosystem by muscle activity. These findings provide a robust scientific basis for identifying novel therapeutic targets.

## 4 Gut-muscle axis crosstalk: mechanisms linking dysbiosis to sarcopenia

### 4.1 Inflammation

Chronic low-grade inflammation is a mild yet persistent systemic inflammatory state that serves as a central pathophysiological link between gut microbiota dysbiosis and age-related sarcopenia (Antuña et al., 2022). With advancing age, the gut microbiota undergoes profound structural alterations, characterized not by unidirectional changes in individual bacterial taxa, but by a broader ecological imbalance, specifically, a decline in anti-inflammatory and protective microbial populations (including *Lactobacillus*, *Bacteroides*, *Prevotella*, and *Faecalibacterium*, a key producer of butyrate) alongside an aberrant expansion of potential pro-inflammatory genera such as *Liminibacter* and *Escherichia-Shigella* (Wang et al., 2025).

This specific microbial dysbiosis promotes systemic inflammation through a dual-pathway mechanism. The depletion of beneficial bacteria, particularly *Faecalibacterium*, results in markedly reduced production of short-chain fatty acids (SCFAs), such as butyrate. SCFAs serve not only as critical energy substrates for maintaining intestinal barrier integrity but also exert potent anti-inflammatory effects by inhibiting histone deacetylases and activating G-protein-coupled receptors including GPR43 and GPR109a. These actions lead to suppression of the NF-κB signaling pathway in both local intestinal and systemic compartments and promote the differentiation of regulatory T cells, thereby enhancing immune tolerance (Chen et al., 2024). Consequently, the loss of *Faecalibacterium* directly impairs endogenous anti-inflammatory capacity. Concurrently, the expansion of Gram-negative bacteria such as Shigella increases the release of pathogen-associated molecular patterns (PAMPs), particularly lipopolysaccharides, which compromise intestinal barrier function and translocate into the bloodstream. These PAMPs activate Toll-like receptors, triggering downstream activation of the NF-κB and NLRP3 inflammasome pathways, ultimately driving sustained elevation of pro-inflammatory cytokines, including IL-1β, IL-6, and CXCL2, in the serum (Cattaneo et al., 2017; Wang et al., 2025).

Ultimately, this persistent inflammatory state exerts a direct detrimental impact on skeletal muscle tissue. Elevated pro-inflammatory cytokines, including TNF-α and IL-6, activate key transcription factors such as NF-κB and STAT3, leading to significant upregulation of the muscle atrophy–associated E3 ubiquitin ligases MuRF1 and Atrogin-1, thereby promoting the ubiquitin-mediated degradation of structural proteins such as myosin heavy chain. Concurrently, these cytokines impair the insulin/IGF-1 signaling pathway by suppressing the Akt/mTOR/S6K1 signaling axis, a central regulator of protein synthesis (Pérez-Baos et al., 2018). The concomitant suppression of anabolic processes and enhancement of catabolic activity collectively drive progressive and sustained loss of muscle mass in older adults.

### 4.2 Microbiota-derived metabolites: key mediators in sarcopenia pathogenesis

The production of key microbial metabolites is profoundly influenced by exogenous factors, particularly diet and pharmaceuticals. Dietary interventions, such as high-fiber diets that provide substrates for fermentation, or polyphenol-rich foods (e.g., berries, green tea) that are metabolized by specific bacteria, can significantly alter the metabolic output of the gut microbiota (Cardona et al., 2013; Sonnenburg and Sonnenburg, 2014). Conversely, drugs like antibiotics can drastically reduce the abundance of SCFA-producing bacteria, while metformin has been reported to increase SCFA levels (Wu et al., 2017). Regarding their stability and role, many of the most biologically active molecules are secondary metabolites. Primary metabolites like SCFAs (e.g., acetate, propionate, butyrate) are relatively stable within the gut lumen but are rapidly absorbed and utilized by host tissues, resulting in a relatively short systemic half-life. It is these secondary metabolites, transformed from dietary components by microbial enzymes, that often serve as the critical signaling molecules in the gut-muscle axis, directly modulating host inflammation, metabolism, and gene expression.

#### 4.2.1 Short chain fatty acids

The SCFAs, which are the primary metabolic products generated by the intestinal microbiota through the fermentation of dietary fibers and other carbohydrates, influence skeletal muscle quality and function via multiple mechanisms (Van et al., 2024). SCFAs, particularly butyrate, exert a multifaceted influence on skeletal muscle homeostasis through direct signaling and indirect systemic effects. The most prominent direct mechanism involves the activation of specific G-protein coupled receptors (e.g., GPR43) on muscle cells, which initiates downstream signaling cascades that converge on the AKT/mTOR pathway to promote protein synthesis, while simultaneously suppressing the FoxO3a/Atrogin-1 axis to inhibit protein degradation (Liu et al., 2024). This dual action is critically supported by evidence that the pro-growth effects of SCFAs are abrogated by mTOR inhibitors like rapamycin. Beyond anabolic signaling, SCFAs also enhance muscle energy metabolism by activating the AMPK/PGC-1α axis, thereby stimulating mitochondrial biogenesis and improving oxidative capacity. The indirect protective effects of SCFAs are equally crucial. Butyrate potently antagonizes chronic, low-grade inflammation, a key driver of sarcopenia, by inhibiting the NF-κB pathway and subsequent production of pro-inflammatory cytokines such as IL-6 (Van et al., 2024). Furthermore, SCFAs are fundamental to maintaining gut health. By upregulating tight junction proteins, butyrate reinforces the intestinal barrier, reducing systemic endotoxemia (as reflected by lower LBP levels) and potentially mitigating gut-derived inflammation that can damage muscle (Qaisar et al., 2025). This improved gut-muscle axis function is further evidenced by the reduction in neuromuscular junction degradation biomarkers like CAF22 following butyrate supplementation (Qaisar et al., 2024b).Critically, this multi-level mechanism is translationally relevant. Preclinical studies in aged mice consistently show that SCFA supplementation improves grip strength and muscle fiber size (Zhu et al., 2024), and these findings are now beginning to be corroborated in human trials. For instance, oral butyrate supplements have been shown to increase muscle mass and physical performance in elderly sarcopenic patients (Rondanelli et al., 2024), providing promising initial support for the therapeutic potential of targeting the gut-muscle axis.

#### 4.2.2 Niacin

Nicotinic acid serves as a precursor for nicotinamide adenine dinucleotide (NAD+). Through metabolomics analysis, Zhang et al. (2025) identified nicotinic acid as a key metabolite produced by Bifidobacterium adolescentis, which is associated with the improvement of muscle mass and function in sarcopenic patients. With advancing age, NAD+ levels progressively decline, and this deficiency is more pronounced in individuals with sarcopenia. Silent information regulator 1 (SIRT1), an NAD+-dependent deacetylase, exhibits activity that is directly influenced by the availability of NAD+ (Khan et al., 2014). SIRT1 activates PGC-1α through deacetylation, promoting mitochondrial biogenesis and functional recovery (Beltrà et al., 2023). This enhances the energy supply to muscle cells and improves muscle function. Furthermore, nicotinic acid upregulates the expression of myogenic genes such as myogenin and MyoD in muscle satellite cells by activating the NAD+/SIRT1 axis, thereby promoting the proliferation and differentiation of these cells and enhancing muscle growth and regeneration (Zhang et al., 2025). Supplementation with nicotinic acid restores NAD+ levels in aged mice to those observed in young mice, while improving mitochondrial function, contractile performance, and reducing the expression of muscle atrophy-related proteins (Zhang et al., 2025). As a potent NAD+ precursor, niacin supplementation (750–1,000 mg/day) restores systemic NAD+ levels in mitochondrial myopathy patients, improving muscle strength and mass (Zhang et al., 2025).

#### 4.2.3 Branched-chain amino acids

Research has demonstrated that branched-chain amino acids (BCAAs) exert a dual and seemingly paradoxical role in the pathogenesis of sarcopenia. On one hand, BCAA supplementation enhances muscle protein synthesis through activation of the mTOR signaling pathway, leading to increased muscle mass and improved muscle function (Ikeda et al., 2020). On the other hand, metabolic dysregulation of BCAAs results in their systemic accumulation, which has been identified as a key contributor to muscle atrophy (Zuo et al., 2025). This apparent contradiction underscores the critical dependence of BCAA biological effects on metabolic homeostasis. Notably, emerging evidence indicates that the gut microbiota plays a central role in regulating BCAA metabolism and maintaining this homeostatic balance.

The gut microbiota plays a critical role in the metabolism of BCAAs. Evidence indicates that increased abundance of Prevotella copri and Bacteroides vulgatus enhances BCAA biosynthesis, whereas reduced levels of Butyrivibrio crossotus and Eubacterium siraeum impair their capacity to mediate BCAA uptake and catabolism (Qiao et al., 2022), a profile that closely aligns with the gut microbial dysbiosis observed in individuals with sarcopenia (Mayer et al., 2024). In the context of microbial imbalance, particularly when BCAA-metabolizing taxa such as *Butyrivibrio crossotus* and *Eubacterium siraeum* are depleted, host BCAA catabolic capacity is diminished, leading to aberrant accumulation of BCAAs and their metabolites, branched-chain α-keto acids (BCKAs), in both systemic circulation and skeletal muscle tissue. Clinical evidence supports this mechanism: in sarcopenia patients, the activity of the rate-limiting enzyme for BCAA degradation, the branched-chain α-keto acid dehydrogenase complex (BCKDH), along with the expression of its upstream activator protein phosphatase Mg^2+^/Mn^2+^-dependent 1K (PPM1K), is significantly downregulated, directly contributing to intracellular BCAA accumulation. Furthermore, when plasma BCAA concentrations reach or exceed 450 μmol/L, the average skeletal muscle mass index (ASMI) declines by 0.21 kg/m^2^, grip strength decreases by 2.3 kg, and gait speed slows by 0.05 m/s (*P* < 0.01) (Zuo et al., 2025). Demonstrating that excessive BCAA accumulation profoundly impairs skeletal muscle mass and function.

The coexistence of BCAA overload and catabolic impairment disrupts the delicate equilibrium between beneficial mTOR signaling activation and detrimental metabolic stress. Chronically elevated BCAA levels drive excessive activation of the mTOR pathway, surpassing its physiological role in promoting protein synthesis and instead triggering insulin resistance through disruption of the insulin signaling cascade and potent suppression of autophagy (Nakamura et al., 2022; Stanciu et al., 2024). Impaired autophagy fails to efficiently remove damaged mitochondria and misfolded proteins, while persistent mTOR overactivation further aggravates mitochondrial dysfunction and oxidative stress. Thus, under conditions of BCAA metabolic dysregulation, the hyperactivated mTOR pathway shifts from a physiological builder of muscle tissue to a pathological destroyer, establishing a self-reinforcing vicious cycle with insulin resistance, autophagy inhibition, and mitochondrial impairment that ultimately culminates in the progressive loss of skeletal muscle mass and function.

In conclusion, future nutritional intervention strategies for sarcopenia should move beyond the simplistic dichotomy of “supplementing” versus “restricting” BCAAs and instead prioritize the restoration of systemic BCAA metabolic homeostasis, with particular emphasis on the role of the gut microbiota. Targeted modulation of the gut microbiota to normalize BCAA metabolism represents a highly promising therapeutic avenue for the prevention and management of sarcopenia.

#### 4.2.4 Lipopolysaccharide

Lipopolysaccharide (LPS), an endotoxin derived from Gram-negative bacterial outer membranes, significantly contributes to sarcopenia pathogenesis through pro-inflammatory and metabolic dysregulation mechanisms. Research has demonstrated that LPS increases the serum concentration of the pro-inflammatory cytokine IL-6, upregulates the expression of Drp1, enhances mitochondrial fission, and reduces total muscle mass (Yu et al., 2024). In C2C12 myotubes, LPS increased the LC3-II/LC3-I ratio, autophagosome formation significantly augmented, and substantially upregulated the expression of Atrogin-1/MAFbx and MuRF1, exacerbating muscle atrophy. Mechanistically, LPS activates the p38 mitogen-activated protein kinase (MAPK) and NF-κB signaling pathways via Toll-like receptor 4 (TLR4). NF-κB activation synergistically regulates the expression of genes involved in the UPS and autophagy (Doyle et al., 2011). Gut dysbiosis increases circulating LPS (Song et al., 2020). Moreover, LPS disrupts the tight junctions of intestinal epithelial cells (Shi et al., 2025), impairing intestinal barrier function and further aggravating muscle atrophy Table 1.

**TABLE 1:** Modulation of sarcopenia pathways by microbial metabolites and related trends.

Microbial metabolites	Physiological function	Modulation of sarcopenia pathways	Trends in sarcopenia	References
SCFAs	Inhibiting the NF-κB signaling pathway	Protection from inflammaging	↓	Hu et al., 2024; Qu et al., 2025; Tang et al., 2022; Wang et al., 2012; Wu et al., 2020; Ye et al., 2024
	Activating PGC-1α	Enhancement of mitochondrial function	↓	
	Increasing expression of IRS-1 gene	Improvement of insulin sensitivity	↓	
	Upregulating Occludin and ZO-1	Enhancement of intestinal barrier integrity	↓	
	Activating AMPK signaling pathway	Improvement of protein synthesis	↓	
Niacin	Activating the SIRT1/PGC-1α axis	Enhancement of mitochondrial biogenesis	↓	Khan et al., 2013; Pirinen et al., 2020; Zhang et al., 2025
	Inducing the conversion of type II to type I muscle fibers	Enhancement of the oxidative metabolic capacity of muscles	↓	
	Inhibiting the FoxO3/Atrogin-1/Murf-1 signaling axis	Suppression of muscle protein breakdown	↓	
Bile acid	Activating FXR and TGR5	Metabolic homeostasis	↓	Fleishman and Kumar, 2024; Yang et al., 2024b
	Inhibiting the association of NLRP3 and pro-Caspase-1	Protection from inflammaging	↓	
Branched-chain amino acids	Disorders of branched-chain amino acid metabolism	Formation of Insulin resistance -autophagy inhibition- mitochondrial damage vicious cycle	↑	Nakamura et al., 2022; Zuo et al., 2025
LPS	Promoting muscle protein degradation	Activating the ubiquitin-proteasome system and autophagy-lysosome system	↑	Doyle et al., 2011; Shi et al., 2025; Song et al., 2020; Yu et al., 2024
	Disrupting the tight junctions of intestinal epithelial cells	Intestinal barrier dysfunction	↑	

↑, increase; ↓, decrease.

### 4.3 Neuroendocrine system

The gut microbiota modulates the neuroendocrine system via the gut-brain axis, constituting a sophisticated and intricately regulated biological pathway that influences skeletal muscle homeostasis. This bidirectional regulatory network exhibits marked sexual dimorphism, potentially contributing to sex-specific differences in susceptibility to sarcopenia and its progression.

On one hand, stress signals, such as elevated cortisol levels, can induce intestinal microbiota dysbiosis and compromise intestinal barrier integrity, thereby triggering systemic inflammatory responses via the LPS-TLR4 signaling pathway and ultimately activating molecular pathways associated with muscle atrophy (Lin et al., 2024). Evidence from mouse models with hypercortisolemia demonstrates a significant reduction in gut microbial diversity, characterized by a marked decrease in beneficial genera including *Bifidobacterium* and *Lactobacillus*, alongside a concomitant increase in potential pathobionts such as Escherichia coli and Streptococcus, further substantiating this mechanistic link (Nie et al., 2024). On the other hand, the gut microbiota exerts positive regulation over anabolic processes through multiple mechanisms: microbial metabolites, particularly SCFAs, have been shown to modulate the secretion of hypothalamic gonadotropin-releasing hormone (GnRH) in a remote manner, thereby enhancing pituitary-gonadal axis function and sustaining physiologically relevant levels of testosterone and estrogen, hormones critical for the maintenance of skeletal muscle mass (Judd et al., 1979; Qiu et al., 2007). Furthermore, Lactobacillus plantarum HL2 has been demonstrated to attenuate ovarian pathological alterations and restore normal secretion of luteinizing hormone, follicle-stimulating hormone, and testosterone, providing compelling experimental support for this regulatory axis (He et al., 2020). Emerging evidence indicates that specific members of the gut microbiota can modulate intestinal immune responses and promote the production of interleukin-13 (IL-13). Notably, enrichment of Prevotella copri has been shown to drive the polarization of naïve CD4+ T cells toward the T helper 2 (Th2) lineage within the gut mucosa, This specific skewing of the adaptive immune response leads to the production and elevation of interleukin-13 (IL-13) in the portal circulation. This cytokine is then transported to the liver via the portal vein, where it activates the IL-13 receptor (IL-13R)-JAK2-STAT6 signaling pathway, thereby triggering robust synthesis of insulin-like growth factor 1 (IGF-1) (Ma et al., 2024). This mechanistic insight directly connects intestinal immune modulation with systemic anabolic signaling essential for muscle growth.

It is noteworthy that the association between gut microbiota and muscle mass is markedly stronger in men than in women. For example, Park et al. (2022) reported in a large-scale population study that in males, the abundance of Roseburia faecis and Haemophilus parainfluenzae, taxa associated with butyrate production and improved insulin sensitivity, was significantly elevated, and gut microbial alpha diversity (assessed using the Shannon index) was positively correlated with skeletal muscle index (SMI). Asaoka et al. (2025) further confirmed that male patients with sarcopenia exhibited significantly reduced alpha diversity (Shannon index, *p* = 0.004), distinct beta diversity profiles, and markedly decreased abundance and detection rates of butyrate-producing taxa such as *Eubacterium eligens* and *Fusicatenibacter*. In contrast, no such significant alterations were observed in female sarcopenia cohorts. Notably, Park’s study, conducted in a middle-aged and younger male cohort (mean age: 45 years), already revealed a robust gut microbiota–muscle mass association, whereas Asaoka et al. (2025) identified an even stronger correlation in elderly men (mean age: 79 years), suggesting that age-related declines in testosterone levels and reductions in butyrate-producing bacteria may act synergistically, via modulation of hypothalamic-pituitary-adrenal (HPA) axis activity and insulin sensitivity, to promote muscle catabolism. This sexual dimorphism may be attributed to multiple underlying mechanisms: testosterone, the primary anabolic hormone in men, and its biosynthetic and signaling pathways, appear to be particularly sensitive to gut microbial status. With advancing age, the natural decline in circulating testosterone, when compounded by depletion of SCFA-producing genera (e.g., *Roseburia faecis*, *Eubacterium*), may exacerbate insulin resistance and low-grade systemic inflammation, thereby accelerating net skeletal muscle protein loss. In contrast, the female immune system typically demonstrates heightened innate inflammatory responsiveness, which may alter the functional weight of the microbiota-immune-IGF-1 regulatory axis or buffer its effects through estrogen-mediated protective mechanisms and other physiological compensatory pathways.

### 4.4 Myokines

The bidirectional crosstalk between skeletal muscle and the intestine involves not only signal reception by skeletal muscle but also its active role as an endocrine organ, capable of releasing specific myokines to modulate intestinal function (Alpuim Costa et al., 2025). This section highlights the pivotal roles of exercise-induced myokines, such as irisin, myostatin (MSTN), and brain-derived neurotrophic factor (BDNF), in the “muscle-to-gut” retrograde signaling axis. These myokines contribute to the maintenance of intestinal homeostasis through coordinated regulation of intestinal barrier integrity, immune-inflammatory responses, and microbial community composition.

Irisin, an exercise-induced myokine, functions as a key mediator in the transmission of anti-inflammatory and protective signals from skeletal muscle to the intestine. Accumulating evidence indicates that Irisin exerts systemic beneficial effects on intestinal homeostasis. In preclinical models of ulcerative colitis and acute pancreatitis, administration of exogenous Irisin has been shown to significantly attenuate intestinal inflammation and tissue injury through inhibition of the MAPK signaling pathway and suppression of apoptotic processes (Huangfu et al., 2021; Ma et al., 2023; Ren et al., 2019). Notably, these protective effects are closely associated with remodeling of the gut microbiota composition, including enrichment of Deferribacteres and reduction of Bacteroides (Huangfu et al., 2021), suggesting that Irisin may mediate its therapeutic benefits via the “immune-microbiota” axis.

Myostatin, a key negative regulator of skeletal muscle growth, exerts significant influence on intestinal health. Studies using MSTN gene knockout pig models have demonstrated a notable phenotype: concomitant with muscle overgrowth, intestinal architecture is markedly improved, characterized by thickening of the muscularis layer of the intestinal wall and upregulation of tight junction proteins, including ZO-1 and Occludin. Importantly, the abundance of SCFA-producing bacteria is significantly increased in the gut (Luo et al., 2023). These findings strongly indicate that MSTN not only governs skeletal muscle development but also, through its absence, may indirectly enhance intestinal microecological balance and mucosal barrier integrity by modulating the secretion profile of myokines or systemic metabolic homeostasis.

Brain-derived neurotrophic factor elucidates the intricate interplay between the neuromuscular system and intestinal function. Beyond its well-documented role at the neuromuscular junction, BDNF originating from skeletal muscle is essential for preserving the integrity of the distal intestinal barrier. BDNF deficiency results in the disrupted expression of tight junction proteins in the colonic epithelium, including ZO-1, Occludin, and Claudin-1, leading to ultrastructural alterations and impaired barrier function, as demonstrated in BDNF knockout (BDNF−/−) mouse models (Li et al., 2018). Furthermore, accumulating evidence indicates that BDNF significantly contributes to the regulation of gut microbiota composition and the maintenance of intestinal homeostasis. Administration of BDNF in mice induces marked remodeling of the gut microbial architecture, characterized by increased alpha diversity, enrichment of beneficial SCFA-producing genera (e.g., *Akkermansia*, *Faecalibacterium*, and *Lactobacillus*), and reduced abundance of potential pathobionts such as *Escherichia coli* and *Staphylococcus* (Li et al., 2018). Collectively, these microbial shifts promote enhanced intestinal health. In summary, muscle-derived BDNF functions as a key endocrine mediator, critically involved in sustaining intestinal barrier integrity and modulating microbial community balance.

**FIGURE 2:**
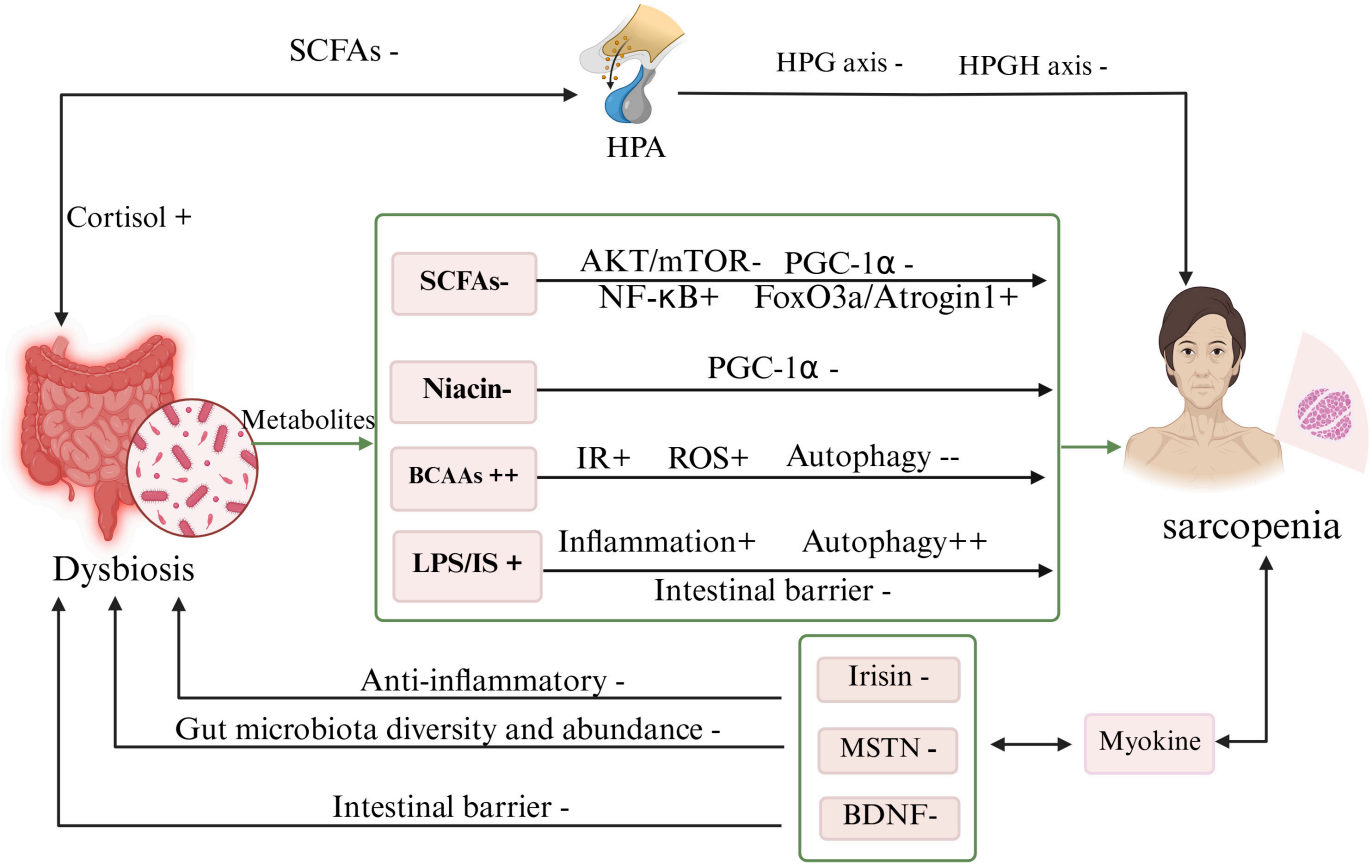
Bidirectional interactions between gut microbiota and skeletal muscle in sarcopenia (Created in BioRender. Lingli, G. (2025) https://BioRender.com/undefined). This schematic illustrates the bidirectional crosstalk between gut microbiota dysbiosis and skeletal muscle dysfunction in sarcopenia. (1) Gut to muscle signaling: SCFAs: Gut dysbiosis leads to a decrease in SCFA-producing bacteria. Reduced SCFA levels inhibit the AKT/mTOR anabolic signaling pathway, suppressing MPS. Concurrently, low SCFAs can activate the NF-κB pathway, exacerbating local and systemic inflammation (P) (Liu et al., 2024; Van et al., 2024). LPS: Impaired intestinal barrier function (−) allows for the translocation of LPS into circulation. LPS activates TLR4 signaling, leading to increased production of pro-inflammatory cytokines (e.g., IL-6, TNF-α) and activation of the UPS and autophagy, thereby promoting muscle proteolysis (P) (Doyle et al., 2011). BCAAs: Dysbiosis may lead to excessive accumulation (++) of BCAAs in circulation. This can exacerbate IR and ROS, while paradoxically inhibiting the initiation of autophagic flux, impairing the clearance of damaged cellular components (C) (Zuo et al., 2025). Niacin: Reduced microbial production of niacin is associated with decreased expression of PGC-1α, a master regulator of mitochondrial biogenesis, contributing to mitochondrial dysfunction and muscle weakness (P/C) (Zhang et al., 2025). Gut-derived SCFAs help regulate the HPG and HPGH axes. Their reduction is linked to decreased secretion of anabolic hormones (e.g., testosterone, IGF-1) (P) (He et al., 2020). Conversely, dysbiosis can promote hyperactivity of the HPA axis, leading to increased cortisol levels, which further suppresses MPS and promotes MPB (C) (Lin et al., 2024). (2) Muscle-to-Gut Signaling via Myokines: Skeletal muscle secretes myokines that directly influence the gut. In sarcopenia, reduced levels of Irisin, MSTN, and muscle-derived BDNF are associated with decreased gut microbiota diversity, impaired intestinal barrier function, and increased inflammation, thereby exacerbating gut dysbiosis and completing the vicious cycle (P) (Huangfu et al., 2021; Li et al., 2018; Luo et al., 2023). +, increase; −, decrease; ++, excessive accumulation or activation; −−, inhibition. (C), Clinical; (P), Preclinical. AKT, Protein Kinase B; BCAA, Branched-Chain Amino Acid; BDNF, Brain-Derived Neurotrophic Factor; HPA, Hypothalamic-Pituitary- Adrenal axis; HPG, Hypothalamic-Pituitary-Gonadal axis; HPGH, Hypothalamic-Pituitary-Growth Hormone axis; IGF-1, Insulin-like Growth Factor 1; LPS, Lipopolysaccharide; MPB, Muscle Protein Breakdown; MPS, Muscle Protein Synthesis; MSTN, Myostatin; mTOR, Mechanistic Target of Rapamycin; NF-κB, Nuclear Factor Kappa-B; PGC-1α, PPARγ Coactivator 1-α; ROS, Reactive Oxygen Species; SCFA, Short-Chain Fatty Acid; TLR4, Toll-Like Receptor 4; TNF-α, Tumor Necrosis Factor-Alpha; UPS, Ubiquitin-Proteasome System.

In summary, skeletal muscle actively participates in the regulation of intestinal function through the secretion of a diverse array of myogenic factors. Irisin primarily mediates anti-inflammatory responses and the modulation of the gut microbiota. The absence of MSTN is closely associated with enhanced intestinal barrier function and improved microbial metabolic profiles. BDNF, on the other hand, regulates the composition of the gut microbiota while directly reinforcing the intestinal physical barrier. Collectively, these factors construct a systemic network through which skeletal muscle influences intestinal health from multiple dimensions, including immune regulation, structural integrity, and microbial balance. This underscores the complexity and precision of the “top-down” regulatory signals originating from skeletal muscle within the gut-muscle axis. This mechanism not only deepens our understanding of the health benefits of exercise but also provides potential molecular targets for developing novel therapeutic strategies for intestinal or muscle-related diseases Figure 2.

## 5 Harnessing the gut-muscle axis: multimodal approaches to combat sarcopenia

### 5.1 Microbiota modulators

Dysbiosis of the gut microbiota is a significant risk factor for the development of sarcopenia in elderly individuals. Consequently, modulating the composition of the gut microbiota has emerged as a critical strategy for mitigating sarcopenia. Studies have demonstrated that oral administration of Lactobacillus P62 (LP), Bifidobacterium P61 (BB), or a combination of both to aged mice can significantly enhance muscle strength and exercise capacity. Further research indicates that the primary mechanism of action involves activating the AKT signaling pathway, promoting muscle protein synthesis, inhibiting FOXO3a and NF-κB activity, reducing muscle catabolism and inflammatory responses, thereby alleviating age-related muscle atrophy (Baek et al., 2023). Additionally, a 16-week single-center randomized double-blind trial revealed that a probiotic supplement containing *Bifidobacterium* and *Lactobacillus* could increase muscle mass, strength, and activities of daily living in elderly men with age-related sarcopenia while concurrently reducing plasma LPS levels (Qaisar et al., 2024a).

Prebiotics, although indigestible by humans, are selectively utilized by intestinal microorganisms, thereby improving the composition and/or activity of the gut microbiota and exerting beneficial effects on human health. Research has shown that prebiotics can selectively stimulate the growth of beneficial bacteria such as *Bifidobacterium* and *Lactobacillus*, while inhibiting the proliferation of harmful bacteria, thus optimizing the intestinal microecological balance. A study involving individuals over 65 years old with frailty syndrome found that continuous oral administration of a prebiotic composed of inulin and fructooligosaccharides for 13 weeks significantly increased grip strength and reduced fatigue scores (Yang et al., 2024a).

Synbiotics, which combine probiotics and prebiotics, have been shown to enhance muscle anabolic processes by regulating the balance of the gut microbiota, inhibiting the growth of harmful bacteria, reducing pro-inflammatory cytokine production, and promoting the generation of SCFAs. For example, a study using kimchi, a fermented product containing *Lactobacillus plantarum* and *Lactobacillus mesenteroides*, fed to mice with malignant adenomas, demonstrated decreased expression and serum levels of the pro-inflammatory IL-6, along with a significant increase in muscle mass by inhibiting the expression of Atrogin-1 and MuRF-1 genes and up-regulating the expression of mitofusin-2 and PGC-1α (An et al., 2019).

Although numerous studies have indicated the potential benefits of probiotics, prebiotics, and synbiotics in ameliorating sarcopenia-related parameters, the current body of clinical evidence remains markedly inconsistent. This heterogeneity may be attributed to several critical factors. First, the therapeutic effects of probiotics are highly strain-specific. Substantial differences in genetic profiles and functional metabolic activities exist among strains (Truong et al., 2017), implying that not all *Lactobacillus* or *Bifidobacterium* strains elicit comparable anti-sarcopenic effects. For example, certain strains, such as *Lactobacillus plantarum* TWK10, have demonstrated efficacy due to their distinct metabolic properties (Chen et al., 2016), whereas others may lack beneficial activity. Second, dosage and treatment duration represent key determinants of intervention outcomes. The wide variability in administered doses (e.g., 10^∧^9–10^∧^11 CFU/day) and intervention periods (ranging from 8 to 24 weeks) across existing studies may partly explain conflicting results; insufficient dosing or inadequate duration may fail to induce sustained modulation of the gut microbiota (Prokopidis et al., 2023). Third, host-specific factors play a pivotal role. Seminal research has established that the composition and baseline stability of an individual’s gut microbiome are fundamental to the successful engraftment and functional activity of probiotic strains (Zmora et al., 2018). Moreover, elderly individuals with sarcopenia frequently use multiple medications—including antibiotics and proton pump inhibitors—and experience age-related immune alterations, both of which can perturb the intestinal microenvironment. These factors may impair probiotic colonization and functionality and, in certain contexts (e.g., following antibiotic exposure), delay the restoration of indigenous microbial communities (Suez et al., 2018).

In summary, future sarcopenia research must move toward precision microbiome interventions through the development of personalized regimens to ensure consistent and reliable therapeutic outcomes.

### 5.2 Fecal microbiota transplantation

Fecal Microbiota Transplantation (FMT), as a therapeutic approach that enhances host health by modulating the gut microbiota, has shown promise in preliminary research in addressing sarcopenia in elderly populations. In a preclinical study, fecal samples from healthy young mice were collected and processed into a microbiota suspension suitable for transplantation. This suspension was subsequently administered to the intestines of aged mice. The results indicated that the FMT-treated elderly mice exhibited significantly improved performance in grip strength and exercise endurance tests compared to the control group. This improvement may be attributed to the significant increase in jejunal microbiota α-diversity induced by FMT, leading to the enrichment of bacterial genera such as *Pseudoscardovia*, *Solobacterium*, *Shuttleworthia*, and *Pseudoraminibacter*, and the modulation of metabolic pathways for carbohydrates, amino acids, and vitamin (Mo et al., 2023). Preliminary clinical evidence suggests that in older adults (≥60 years) with sarcopenia, a regimen of FMT delivered via a nasojejunal tube, coupled with resistance training, may be associated with improved outcomes. Reported benefits in initial studies include an approximate 15% increase in complete remission rates, a rise in the appendicular skeletal muscle index of around 0.1 kg/m^2^, and reductions in serum inflammatory markers such as IL-6, TNF-α, and CRP. Further investigations have revealed that FMT increases the α-diversity of the intestinal microbiota and elevates the abundance of butyrate-producing genera, including *Faecalibacterium*, *Roseburia*, *Dorea*, *Coprococcus*, *Blautia*, and *Agathobacter*. Conversely, the abundances of *Veillonella* and *Erysipelotrichaceae*, both of which exhibit a strong positive correlation with TNF-α (*r* = 0.963), are reduced (Yang et al., 2025). These findings suggest that the therapeutic mechanism may be closely associated with the remodeling of the “butyrate-producing bacteria–short-chain fatty acids–anti-inflammatory–intestinal barrier” axis. Age-related dysbiosis triggers a pathological cascade from the gut to muscle: it compromises the intestinal barrier (reducing goblet cells, Muc-2, and tight junction proteins), leading to LPS translocation, NLRP3-mediated chronic inflammation, and ultimately, suppression of muscle protein synthesis and sarcopenia (Mo et al., 2023). Conversely, FMT from young donors interrupts this cascade by reconstructing a beneficial microbiota (e.g., increasing *Akkermansia* and *Lactobacillus*). This intervention concurrently restores barrier integrity, reduces systemic LPS and inflammation, and ameliorates muscle mitochondrial decline, thereby preserving muscle function (Diao et al., 2018; Mo et al., 2023).

However, it is crucial to interpret these encouraging findings with caution, as the current body of evidence is constrained by several important limitations. Many clinical studies, including the aforementioned preliminary trials, are characterized by small sample sizes and short follow-up durations, which limit the statistical power and the ability to assess the long-term efficacy and stability of FMT interventions. Furthermore, FMT is not without potential risks. Procedural concerns and the possibility of transferring pathogenic or antibiotic-resistant organisms, as evidenced by severe adverse events in immunocompromised patients (DeFilipp et al., 2019), underscore the necessity for rigorous donor screening and safety monitoring.

It is important to recognize that the complete prevention or effective management of long-term dysbiosis recurrence following fecal microbiota transplantation (FMT) continues to represent a significant unmet clinical challenge. FMT fundamentally functions as a single-event “ecological transplantation”; however, the stability of the transplanted microbial community is frequently compromised by ongoing host-specific factors, dietary patterns, medication use, and environmental influences. Furthermore, the limited colonization capacity and relatively short half-life of transplanted bacterial strains further hinder the long-term persistence of the introduced microbiota. In addition, prolonged and repeated administration of whole-community FMT may pose a risk of transmitting antibiotic-resistant pathogens or other harmful microorganisms. Therefore, addressing this challenge extends beyond the scope of individual studies and represents a priority area that requires sustained interdisciplinary research and collaborative efforts across the field.

### 5.3 Nutritional approach

Recent studies suggest that dietary nutrients specifically targeting skeletal muscle, consisting of hydroxymethyl butyrate (HMB), carnosine, magnesium, butyrate, and lactoferrin, may significantly enhance muscle strength, increase muscle mass, and improve the Short Physical Performance Battery (SPPB) score in elderly patients suffering from sarcopenia. Furthermore, these dietary supplements have been shown to reduce plasma levels of CRP and TNF-α, both of which are established markers of inflammation, as well as zonulin, an indicator of intestinal permeability. The reduction in these biomarkers suggests that the dietary supplement possesses anti-inflammatory properties and may contribute to the restoration of intestinal barrier function (Rondanelli et al., 2024). Research has indicated that HMB, a metabolite derived from leucine, enhances muscle mass and strength by promoting protein synthesis and facilitating energy metabolism (Martínez-Arnau et al., 2020). In addition, carnosine is composed of β-alanine and L-histidine, and supplementation of β-alanine can directly increase the carnosine reserve in skeletal muscle and enhancing its mass and function (Furst et al., 2018). In addition, butyrate and lactoferrin in this dietary supplement have been shown to inhibit inflammatory responses by regulating the NF-kB signaling pathway (Frioni et al., 2014). Recent studies have also found that butyrate reduces muscle atrophy by repairing the neuromuscular junction (Qaisar et al., 2024b).

Another randomized double-blind controlled trial showed that, compared with the placebo group with the same caloric and flavor, the group of special medical use nutrition supplement consisting of omega-3 fatty acids, leucine and probiotics Lactobacillus paracasei PS23 significantly improved Tinetti score, SPPB score and grip strength after 2 months (Rondanelli et al., 2022). This special medical use nutrition supplement also employs a multi-target therapeutic strategy to tackle age-related sarcopenia. Notably, older adults with sarcopenia typically consume lower levels of omega-3 polyunsaturated fatty acids, which promote muscle synthesis, and their intake is positively associated with secondary outcomes such as SF-36 psychological scores and Sarcopenia Quality of Life Questionnaire (SarQoL). Therefore, omega-3 fatty acids are regarded as an important potential target for the treatment of muscle atrophy, and the daily supplementation of 0.7g–3.36 g of omega-3 fatty acids is currently recommended for the elderly (Dupont et al., 2023; Rondanelli et al., 2022).

### 5.4 Exercise

Exercise intervention is a cornerstone strategy in the management of sarcopenia, particularly resistance training and aerobic exercise, which have been shown to significantly enhance muscle mass and function in patients with sarcopenia (Millan-Domingo et al., 2024). An 8-week randomized controlled trial examined the effects of kettlebell training on elderly female sarcopenia patients. The results demonstrated that kettlebell training markedly improved muscle mass, grip strength, and lung function, while reducing levels of the chronic low-grade inflammation marker CRP. These improvements persisted for up to 4 weeks post-training cessation *(Chen et al., 2018)*. Another study involving sarcopenic patients aged 80–99 years found that 12 weeks of combined resistance and balance training significantly increased grip strength and back muscle strength (Liang et al., 2020).

Exercise has been shown to directly enhance muscle mass, strength, and physical fitness in sarcopenia patients by modulating the gut microbiota. Exercise training alters the composition of the gut microbiota, reducing the abundance of Clostridium bacteria, especially Blautia bacteria that promote the release of pro-inflammatory cytokine TNF-α. Simultaneously, exercise increases the abundance of *Bacteroidetes*, *Actinobacteria*, and *Bifidobacterium* species with anti-inflammatory properties (Xie and Huang, 2024). Evidence from plasma inflammatory marker assessments indicates that exercise reduces levels of LBP, TNF-α, and CRP through its regulatory effects on the gut microbiota (Motiani et al., 2020). Studies examining the impact of different exercise intensities on gut microbiota and muscle function suggest that long-term high-intensity training may alter microbial composition, specifically decreasing bacteria associated with SCFAs production while increasing those involved in inflammatory processes. Consequently, prolonged high-intensity exercise may increase intestinal permeability and elevate plasma LPS levels, triggering inflammatory responses within skeletal muscles and impacting their function. In contrast, short-term moderate-intensity training appears beneficial for enhancing SCFA-producing bacterial populations and improving physical activity capacity; however, this positive effect tends to diminish following training cessation (Gao and Zhang, 2024). Therefore, it is recommended to maintain a regimen of long-term moderate-intensity exercise to better regulate gut microbiota, reduce muscle inflammation, improve mitochondrial function, and ultimately enhance muscle mass and strength, as well as overall physical function Table 2.

**TABLE 2:** Therapy approaches targeting gut microbiota for attenuating sarcopenia.

Therapeutic strategy	Species	Intervention duration	Outcome	References
**Probiotic supplementation**					
Probiotic	18-month- old male mice	Lactobacillus paracasei P62 (LP), 8 weeks	Grip strength, treadmill distance and running time↑: BL > Lp > Bb FOXO3a, NF-Kb, MuRF1, MAFbx: LP↓, BB↓, BL↓ TNF-α, IL-6: LP↓, BB↓, BL↓ PGC-1α, SIRT1, MyHC: LP↑, BB↑, BL↑	Baek et al., 2023
		Bifidobacterium bifidum P61 (BP), 8 weeks		
		LP and BP (1:4) mix (BL), 8 weeks		
Prebiotic capsules	Men, sarcopenia (>65 years)	Probiotic capsules (bifidobacterial, Streptococcus thermophilus DSM 24731, and lactobacilli), 16 weeks	HGS↑ GSMI↑ Plasma zonulin level↓ SarQoL↑	Qaisar et al., 2024a
Prebiotic blend	Elderly individuals, frailty or pre-frailty (≥65 years)	Prebiotic blend (inulin and oligofructose), 15 g/d, 12 weeks	Walking speed↑, Grip strength↑	Yang et al., 2024a
**FMT**				
FMT	Aged rats, female, 8 weeks	Fecal supernatants from young donor rats (yFMT), 1 ml/d, 8 weeks	Skeletal muscle mass: yFMT↑oFMT↓ Muscle strength and function: yFMT↑FoFMT↓ Satellite cells: yFMT > oFMT MyoD, myogenin, IGF-1: yFMTF-1n, ↓ Atrogin-1, MuRF, myostatin: yFMT↓, oFMT↑	Mo et al., 2023
		Fecal supernatants from old donor rats (oFMT), 1 ml/d, 8 weeks		
	Elderly people, sarcopenia (≥60 years)	RT group, structured RT-based exercise program, 2–3 sessions per week, 8 weeks	FMT+RT group relative to the RT group: ASMI↑, HGS↑, 5R-STS ↓, Walking speed↑, ALB↑, Hb↑, IL6, ↓ TNF↓	Yang et al., 2025
		FMT + RT group, Fecal supernatants from yong donor, donor fecal microbiota was infused into the proximal jejunum via a nasojejunal tube for 6 consecutive days.		
**Nutritional approaches**				
Dietary supplement	Sarcopenic patients, (55–85 years old)	Experimental formula: calcium hydroxymethyl butyrate 1,500 mg, l-carnosine 125 mg, Lactoferrin 50 mg, Sodium butyrate 250 mg, Magnesium 150 mg), twice daily, 12 weeks	Running distance↑iGrip strength↑tSPPB↑PSMI↑ CRP, zonulin, TNF-α:↓	Rondanelli et al., 2024
Leucine supplement	Elderly people, (≥65 years)	Orally administering leucine with water or juice at a dosage of 3 g per serving, twice daily, 13 weeks	Expiratory muscle strength↑ Walking speed↑	Martínez-Arnau et al., 2020
A novel food	Sarcopenic patients, (aged ≥ 55)	Orally administering a novel food (Leucine, Omega-3 Fatty Acids and Probiotic Lactobacillus), 8 weeks	ALM→ Tinetti scale score↑ SPPB total score↑ HGS↑	Rondanelli et al., 2022
**Exercise therapy**				
Kettle bell training	Elderly women, sarcopenia aged 65–75 years	Kettle bell training, twice a week, 8 weeks	Muscle strength↑ Pulmonary function↑ Hs-CRP, IL-6, TNF-α :↓	Chen et al., 2018
Mixed exercise program	Older patients, sarcopenia, aged 80–99 years	Mixed exercise program (balance and resistance exercise), twice-weekly, 12 weeks	ADL↑, Gait speed↑ Handgrip strength↑ SPPB score↑ Number of fallers↓	Liang et al., 2020

↑, increase; ↓, decrease; →, no change.

## 6 Conclusion and future perspectives

As the global population continues to age at an accelerating pace, sarcopenia has become an increasingly critical public health issue, demanding a shift in perspective that transcends the limitations of traditional therapeutic strategies. This review systematically synthesizes accumulating evidence from recent years to propose that the gut–muscle axis plays a central regulatory role in the pathophysiological mechanisms underlying age-related muscle decline. We provide a comprehensive analysis of this intricate bidirectional crosstalk: Dysbiosis of the gut microbiota contributes to the onset and progression of sarcopenia through multiple pathways, including disruption of systemic immune homeostasis (e.g., elevated circulating lipopolysaccharide and pro-inflammatory cytokines), impairment of anabolic signaling (mediated by reduced short-chain fatty acid production), and dysregulation of neuroendocrine functions. Conversely, skeletal muscle exerts reciprocal modulation on gut microbial composition and intestinal mucosal barrier integrity via the secretion of myokines.

This mechanistic insight opens the door to practical, microbiota-targeted applications with substantial clinical potential. The field is now poised to transition from observational associations to targeted interventions, with several key translational priorities emerging. First, the identification of specific microbial signatures, such as depletion of *Faecalibacterium* and an elevated Firmicutes-to-Bacteroidetes (F/B) ratio, provides a foundation for developing non-invasive biomarkers of gut dysbiosis. These biomarkers could be integrated into routine geriatric evaluations to enable early risk screening and longitudinal monitoring of sarcopenia progression. Second, modulation of the gut microbiota represents a promising therapeutic frontier. While FMT requires further protocol standardization, more immediately feasible strategies include the use of targeted probiotic formulations, such as combinations of SCFA-producing strains with barrier-protective species, and precision prebiotics designed to selectively nourish depleted commensal populations. Moreover, postbiotic supplementation, including encapsulated butyrate or other purified microbial metabolites, offers a pharmaceutically tractable means of delivering key gut-derived signaling molecules directly to the musculoskeletal system.

Building upon these interventions, the inherent interindividual variability of the gut microbiota underscores the imperative for personalized therapeutic strategies. Moving beyond a one-size-fits-all approach, baseline microbiome profiling can serve as a decisive tool to guide intervention selection. For instance, elderly individuals exhibiting a profound depletion of SCFA-producing genera (e.g., *Faecalibacterium*, *Roseburia*) might preferentially benefit from precision prebiotics tailored to stimulate these specific taxa. Conversely, those with a microbial signature dominated by pro-inflammatory pathobionts and a compromised gut barrier may respond better to a combined regimen of anti-inflammatory probiotics and barrier-strengthening postbiotics. Furthermore, the dynamic nature of the gut ecosystem calls for longitudinal monitoring of microbial shifts in response to interventions, allowing for real-time adjustment of therapeutic protocols. This personalized framework is particularly crucial in the geriatric population, where diverse dietary habits, polypharmacy, and comorbid conditions significantly shape the gut microbiota and its response to treatment. Integrating microbiome data with these clinical variables will be key to developing effective, safe, and tailored management plans for sarcopenia.

It is worth noting that nutritional approaches and exercise can also be considered as interventions targeting the gut microbiota. Specific dietary patterns, such as those rich in dietary fiber, polyphenols, and fermented foods, have been shown to improve the composition and function of the gut microbiota, thereby having a positive impact on muscle health. Similarly, regular exercise not only directly enhances muscle strength and function but also indirectly promotes muscle health by modulating the diversity and metabolic activities of the gut microbiota. These lifestyle modifications can be combined with microbiota-targeted therapies to achieve a more comprehensive therapeutic effect.

Despite the compelling evidence linking the gut–muscle axis to sarcopenia, this review acknowledges several limitations in the current body of evidence that must be addressed to advance the field. First, the causal relationship remains inadequately defined, as the majority of human studies are observational in nature. Second, our mechanistic understanding is largely derived from reductionist models, such as in vitro cultures or single-species animal studies, leaving the complex interplay of these pathways in the human physiological context poorly understood. Finally, existing interventional trials, including those on FMT, are often constrained by small sample sizes and short follow-up durations, hindering the assessment of long-term efficacy and safety.

To overcome these limitations, future research should prioritize the following directions. To establish causality, the field requires large-scale, prospective cohort studies to delineate temporal and dose-response relationships, complemented by humanized animal models that recapitulate the gut microbiota of aging individuals for direct mechanistic validation. To bridge mechanistic gaps, the integration of multi-omics data (e.g., metagenomics, metabolomics) with clinical phenotypes using advanced bioinformatics and machine learning is essential. This approach will not only decipher complex host-microbe interactions but also empower the development of reliable, non-invasive microbial biomarkers for early diagnosis and risk stratification. Ultimately, for successful clinical translation, research must focus on developing safe, effective, and personalized microbiota-targeted therapies. This includes refining FMT protocols, engineering defined next-generation probiotics and precision prebiotics, and exploring the therapeutic potential of purified postbiotics. By systematically addressing these challenges, the gut–muscle axis holds immense promise for delivering innovative strategies to promote musculoskeletal health throughout aging.

## Glossary

MyoD: myoblast determination protein 1
SCFAs: short-chain fatty acids
MPB: muscle protein breakdown
MPS: muscle protein synthesis
GSK3β: glycogen synthase kinase-3β
UPS: ubiquitin-proteasome system
TGF-β: transforming growth factor-β
SASP: senescence-associated secretory phenotype
IMAT: intermuscular adipose tissue
NLRP3: NOD-like receptor protein 3
NF-κB: nuclear factor kappa-light-chain-enhancer of activated B cells
TNF-α: tumor necrosis factor
UPS: ubiquitin-proteasome system
p38 MAPK: p38 mitogen-activated protein kinase
MSTN: myostatin
Atrogin-1: muscle atrophy f-box
MuRF1: muscle RING-finger protein-1
mTOR: mammalian target of rapamycin
WS: walking speed
ALM: appendicular lean mass
GS: grip strength
FoxO3A: forkhead box O3A
IRS-1: insulin receptor 1
PGC-1α: peroxisome proliferator-activated receptor-gamma coactivator 1-alpha
GPR43: G protein-coupled receptor 43
ZO-1: zonula occludens-1
BCAAs: branched-chain amino acids
LPS: lipopolysaccharide
DRP1: dynamin-related protein 1
p38 MAPK: p38 mitogen activated protein kinase
IS: indoxyl sulfate
CKD: chronic kidney disease
SMI: skeletal muscle index
HGS: hand grip strength
ROS: reactive oxygen species
IL-6: interleukin 6
NLRP3: NOD-like receptor protein 3
TLR4: toll-like receptor 4
CASP9: Caspase9
CASP3: Caspase3
PINK1: PTEN induced putative kinase 1
parkin: parkin E3 ubiquitin protein ligase
PI3K: phosphoinositide 3-kinase
AKT: protein kinase B
IGF1: insulin-like growth factor 1
HPG: hypothalamic-pituitary-gonadal
IR: insulin resistance
IRS1: rnsulin receptor 1
Murf-1: muscle RING-finger protein-1
MAFbx: muscle atrophy F-box protein
FMT: fecal microbiota transplantation
IFN-γ: interferon gamma
HMB: hydroxymethyl butyrate
SPPB: short physical performance battery
CRP: C-reactive protein
SarQoL: sarcopenia quality of life
LBP: lipopolysaccharide-binding protein
SIRT1: silencer of cytokinesis 1
TFAM: mitochondrial transcription factor A
AChRs: acetylcholine receptors
GH: growth hormone
ATF4: activate transcription factor 4
BNIP3: Bcl-2/adenovirus E1B 19-kDa-interacting protein 3
NMJ: neuromuscular junction
HPA: hypothalamic-pituitary-adrenal
AMPK: AMP-activated protein kinase
NRF1/2: nuclear factor E2-related factor1/2
RANTES: regulated upon activation, normal T cell expressed and secreted
MCP-1: monocyte chemoattractant protein-1
BCAAs: branched-chain amino acids
BDNF: brain-derived neurotrophic factor
Mfn-2: mitofusin-2
SF-36: 36-Item Short Form Health Survey
